# Virological Characteristics of Acute Hepatitis B in Eastern India: Critical Differences with Chronic Infection

**DOI:** 10.1371/journal.pone.0141741

**Published:** 2015-11-16

**Authors:** Neelakshi Sarkar, Ananya Pal, Dipanwita Das, Debraj Saha, Avik Biswas, Bhaswati Bandopadhayay, Mandira Chakraborti, Mrinmoy Ghosh, Runu Chakravarty

**Affiliations:** 1 ICMR Virus Unit, Kolkata, ID & BG Hospital Campus, Kolkata, West Bengal, India; 2 Department of Virology, Calcutta School of Tropical Medicine, Kolkata, West Bengal, India; 3 Department of Medicine, ID & BG Hospital Campus, Kolkata, West Bengal, India; Saint Louis University, UNITED STATES

## Abstract

Hepatitis B Virus (HBV) manifests high genetic variability and is classifiable into ten genotypes (A-J). HBV infection can lead to variable clinical outcomes, ranging from self-limiting acute hepatitis to active chronic hepatitis, cirrhosis and hepatocellular carcinoma. The present study characterizes HBV strains circulating among patients with acute (AHB) and chronic HBV infection (CHB). Among a total of 653 HBsAg positive cases, 40 manifested acute infection. After sequencing the surface(S), basal core promoter/pre-core(BCP/PC) and the X gene regions, phylogenetic tree was constructed using MEGA4 by neighbor-joining method. Statistical robustness was established with bootstrap analysis. Nucleotide diversity was determined by Shannon entropy per site using the Entropy program of the Los Alamos National Laboratories. Analyses of acute patients revealed that HBV/D2 is the major circulating sub-genotype and commonly associated with sexual promiscuity and the age group between15-30 years. Comparison of AHB and CHB patients revealed that HBeAg positivity, ALT levels and genotype D were significantly high in AHB, whereas CHB patients were predominantly male, had a high viral load, and were commonly associated with genotype C. The frequencies of mutations in the S, BCP/PC, and X gene were low in AHB as compared to CHB. Drug resistant mutations were not detectable in the polymerase gene of AHB. Average nucleotide diversity in AHB was considerably low as compared to CHB. Further, the highest average ΔH (average difference in entropy between chronic and acute infection) was observed in the BCP/PC region implying that this region was most vulnerable to mutations upon HBV persistence, especially in case of genotype C. Additionally, among all substitutions, the A1762T and G1764A BCP mutations were the strongest indicators of chronicity. In conclusion, the study exhibits a general portrait of HBV strains circulating among acute hepatitis B patients in Eastern India and their intricate differences with chronic patients which should be useful from the clinical point of view.

## Introduction

Hepatitis B virus (HBV) is a global health threat affecting about 350 million people across the world [1]. HBV leads to a wide spectrum of clinical presentations ranging from acute hepatitis, asymptomatic chronic carrier state and chronic hepatitis B (CHB) with progression to liver cirrhosis and hepatocellular carcinoma. In approximately 95% of adults, exposure to HBV leads to an acute infection which usually gets resolved in about 6 months without long-term consequences, whereas the remaining 5–10% fails to control the viral infection, leading to chronic illness [2].

Studies on acute HBV infection are particularly significant since it informs us about newly emerging viral strains [3] apart from also shedding light on the prominent modes by which adult infection is transmitted [4]. Further, it also highlights the mutations in the HBV genome which are transmissible and hence provide extremely important information in the attempts to contain HBV spread [5].

A recent study reported drug resistant mutations in the HBV polymerase gene among acute patients and it thus reveals that certain treatment resistant mutations in HBV are transmissible [4]. Another study from China reports that pedicure in bath centers surpasses all other risk factors in acute HBV transmission [5]. Interestingly, an early study on acute patients in Japan revealed that genotype A has been spreading throughout the country, which had been transmitted from Europe, United States, Philippines and India [6].

India has a high proportion of HBV associated chronic carriers and extensive studies have been carried out on them [7]. However, in spite of its prominent importance, acute infection in India remains largely un-characterized. Interestingly, there is only one report from India which describes the HBV genotypes during acute infection based on traditional PCR-RFLP and TSP-PCR [8]; however molecular characterization was not done. A previous study by Singla et al. on chronic hepatitis patients from India showed that drug resistant mutations in the polymerase gene are most frequent in genotype D [9]. Additionally, they reported the presence of these mutations in treatment-naïve individuals. On account of the predominance of genotype D in the Indian subcontinent and an extensive use of antiretroviral drugs, screening of such mutations in acute patients from India becomes extremely important for monitoring the possible transmission of drug resistant mutants. Thus, the present study characterizes HBV strains circulating among acute patients from Eastern India and compares it with HBV strains from chronic patients belonging to the same population.

## Methods and Materials

### Ethics Statement

This work was a part of the study approved by ‘‘The Institutional Ethical Committee, National Institute of Cholera and Enteric Diseases (ICMR)”. Written informed consent was obtained from all the study participants in their native language.

### Study subjects

The patients included in this study were referred from Infectious Disease and Beliaghata General Hospital, Kolkata and School of Tropical Medicine, Kolkata to our unit for HBV DNA testing. Sera from 40 patients with acute Hepatitis B, screened from a pool of 653 HBsAg positive cases, collected and processed over a period of 4 years, (2008–2011) were used in the current study. An acute viral hepatitis case is defined as a person having an acute illness with a discrete onset of any sign or symptom (eg. Fever, malaise, headache, anorexia, vomiting, diarrhea, nausea, abdominal pain) and either jaundice or elevated serum Alanine aminotransferase (ALT) levels higher than 100 IU on at least two occasions during a week without any history of pre-existing liver disease (UNCDC, 2012). Thus, the criteria selected for screening acute hepatitis B were: (1) Patients having history of HBV infection for less than 6 months (2) Positive for Anti-HBc-IgM (>600 Paul Elrich units) and (3) high ALT (>5ULN) and bilirubin (>5ULN). Signed informed consents were obtained from the study subjects. The study was a part of the project entitled “studies on HBV Genotypes in Eastern India” and was approved by the Institute Ethics Committee. For all patients, there was no evidence for concomitant HCV, HDV or HIV infection, metastatic or autoimmune liver disease.

### Serological Assays

Detection of Hepatitis B surface antigen (HBsAg), Hepatitis B e antigen (HBeAg), antibody against Hepatitis B core antigen (antiHBc-IgM) and antibody against Hepatitis B e antigen (anti-Hbe) were tested by commercial EIA kits (Diasorin, S.P.A., Saluggia, Italy) according to manufacturer’s instructions.

### DNA isolation, HBV DNA detection & determination of HBV genotypes/sub-genotypes

DNA was extracted from 200 μl of plasma by using the QIAamp DNA Blood MiniKit (QIAgen, Hilden, Germany). HBV DNA was detected by nested-PCR amplification from the surface gene region as reported earlier [10]. Guidelines by Kwok and Higuchi were strictly followed for avoiding false positive results in PCR [11].

The amplified surface gene region of 429 base pairs was sequenced directly using Prism Big Dye kit in ABI 3130xl Genetic Analyzer (Applied Biosystems, Foster City, CA, USA). HBV genotypes and sub-genotypes were analyzed by phylogenetic analysis with Mega 4, using the neighbor-joining method [12]. Phylogenetic groups were analyzed by the bootstrap test with 1000 replicates.

### Amplification and sequence analysis of surface gene, x gene, basal core promoter and pre-core gene

The small surface gene, basal core promoter, the pre-core region and the x gene were amplified as described earlier [13,14]. Amplified regions were sequenced directly using Prism BigDye kit in ABI 3130xl Genetic Analyzer (Applied Biosystems, Foster City, CA, USA) and analyzed using Bioedit version 7.1.9. Sequence substitutions were analyzed by aligning study sequences with Genbank retrieved sequences. The sequence data of these acute patients were compared with already reported sequences of chronic patients belonging to the same population and the same time-frame. The respective sequences of the surface gene region [15], x gene [14], basal core promoter [16] and the pre-core region [16] of chronic patients were downloaded from Genbank, screened and analyzed.

The Genbank accession numbers of acute sequences are: KT235585-KT235614 (whole small surface gene sequence), KT235615-KT235639 (X gene sequences) KT259233-KT259269 (BCP/PC) KT259223-KT259232 (partial surface).

### Calculation of Genetic Diversity

Genetic diversity was determined from nucleotide diversity calculated by Shannon entropy per site from amplified gene regions using the Entropy program of the Los Alamos National Laboratories website (/http://www.hiv.lanl.gov/content/sequence/ENTROPY/entropy.htmlS).The genetic diversity of acute sequences (query sequences) of the current study was compared with that of chronic sequences (background sequences)[14, 15, 16]. The total difference in entropy (ΔH) between chronic and acute sequences was divided by the length of the sequence (no. of nucleotides) to determine the average difference in entropy per site.

### Quantification of HBVDNA

HBV DNA was quantified by TaqMan based real time PCR assay in Applied Biosystems SDS7000 (Foster City, CA, USA) using W.H.O. prescribed standards (NIBSC, South Mimms, UK) as done earlier [17, 18].

### Statistical analysis

Comparisons of continuous variables between groups were done by unpaired t test using the Graphpad Prism (version 4.0.3). Categorical variables were analyzed by the chi-square test or Fisher exact test, as appropriate using Epi Info software (CDC, USA). All p-values were 2-tailed and P-value <0.05 was considered to be significant.

## Results

### Prevalence, Modes of Transmission, Age and HBV Genotypes

**Fig 1:**
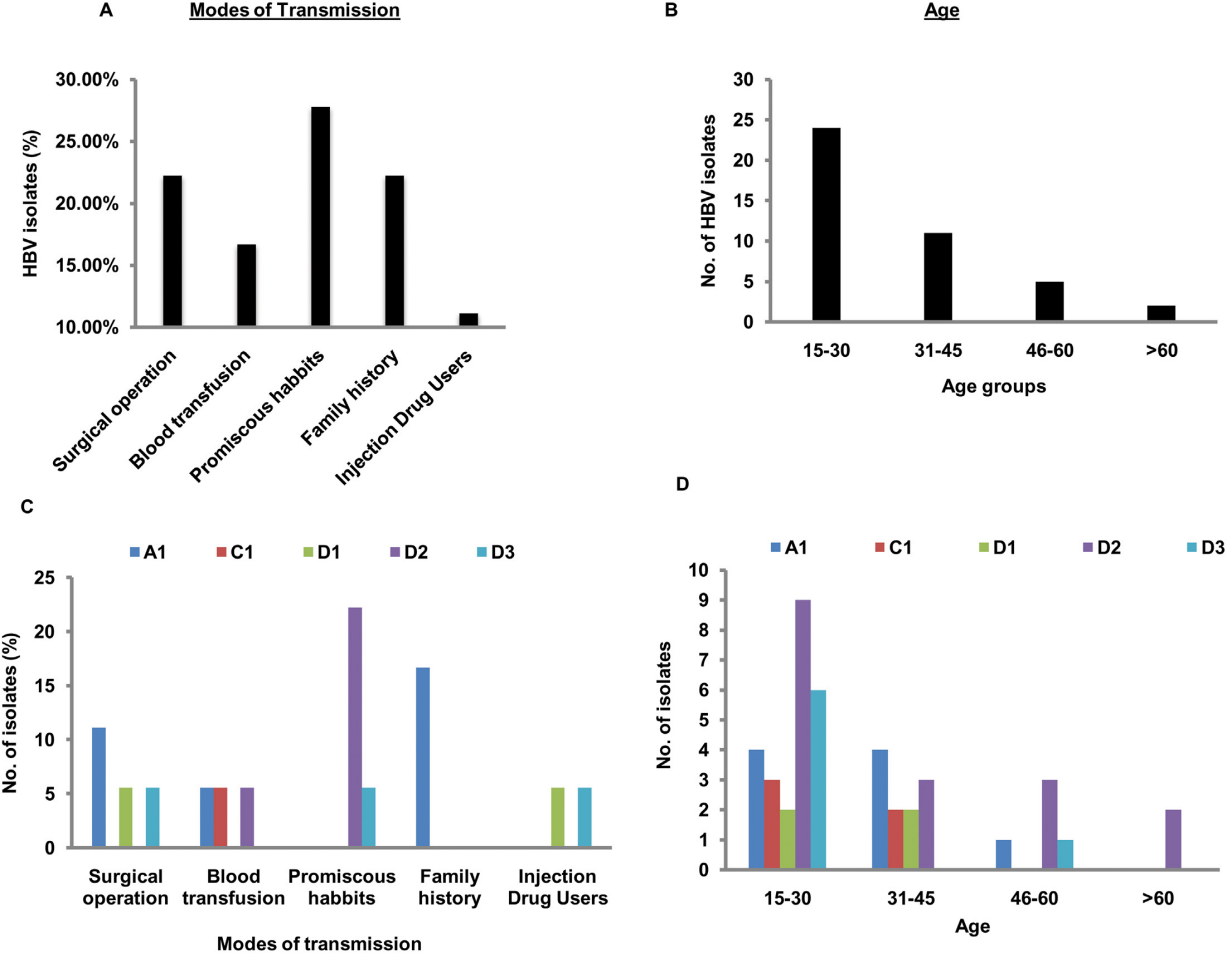
Association of HBV occurrence with the modes of transmission, patients’ age groups and HBV genotypes among samples included in the study. (A) Sexual promiscuity is the major mode by which HBV is transmitted in the study population. (B) Occurrence of HBV is highest in the age bracket between 15 to 30 years. (C) HBV/D2 is the dominant sub-genotype and is associated majorly with sexual promiscuity. (D) Occurrence of HBV/D2 is highest in the age bracket between 15 to 30 years.

Out of 653 HBsAg positive patients who were screened, only 40 patients (6.12%) were found to have acute hepatitis. Of the 40 acute patients, data regarding the modes of transmission of HBV was obtainable from only 18 patients (45%). The rest (55%) either declined to reveal or were unaware of their transmission route. Sexual promiscuity (27.78%) was found to be the major means through which HBV was transmitted in our study population. This was closely followed by familial transmission (22.22%) and transmission during surgical operation (22.22%) (Fig 1A). Notably, patients with acute HBV infection mostly belonged to an age group of 15 to 30 years. There were no acute patients under 15 years of age and the number of patients gradually decreased with increasing age (Fig 1B). Further analysis of genotypes and its association with the modes of transmission revealed that HBV/D2 was the most prevalent sub-genotype which was highly associated with sexual promiscuity (Fig 1C) and notably HBV/D2 was also predominantly associated with the age group of 15 to 30 years (Fig 1D). Interestingly, HBV/A1 is the only sub-genotype circulating among patients who had acquired the infection through familial transmission.

### Biochemical parameters and HBV DNA load

**Fig 2:**
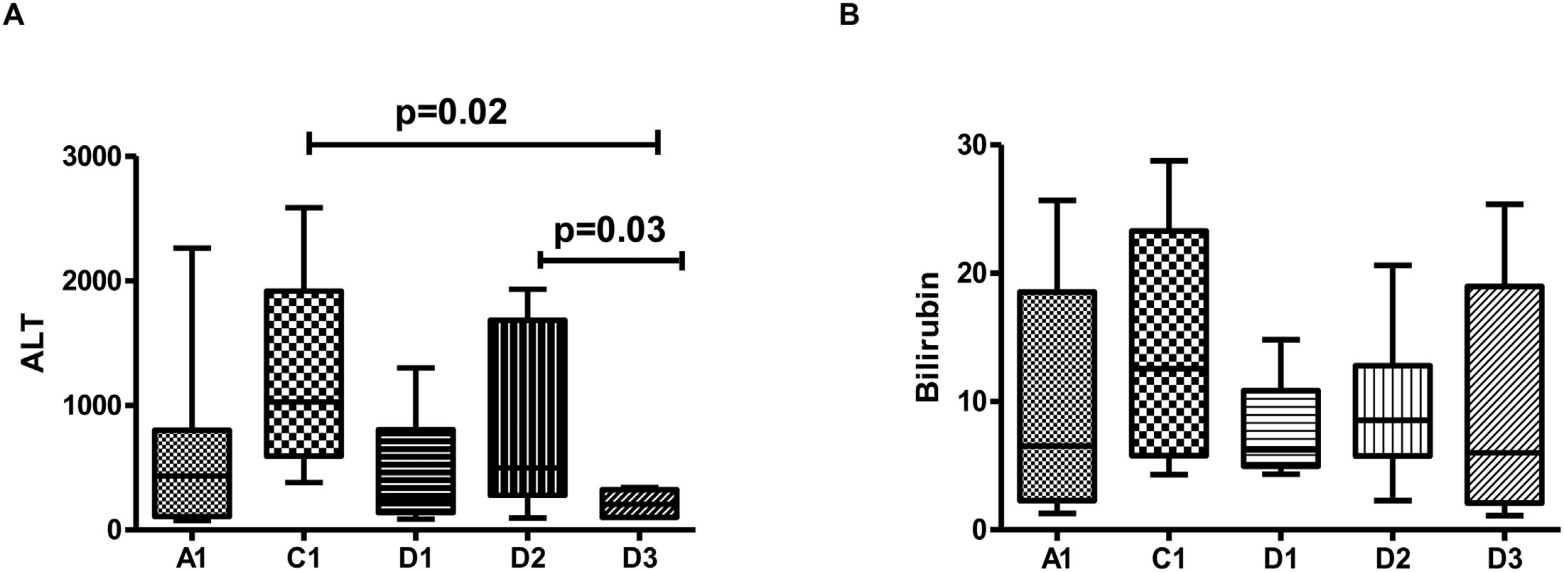
Analysis of the association of HBV sub-genotypes with biochemical parameters. Median (A) ALT (IU/liter) and (B) bilirubin (IU/liter) levels in patients vary with circulating HBV sub-genotypes. Only significant differences (p<0.05) have been marked in the figure.

The ALT levels in our study population ranged from 380 IU/liter to 2280 IU/liter with a median ALT of 400 IU/liter. The levels of bilirubin ranged from 5 mg/dl to 28.75 mg/dl with a median bilirubin of 6.4 mg/dl. The ALT levels varied with HBV sub-genotypes, with the highest ALT in HBV/C1 followed closely by HBV/D2. ALT in HBV/D3 was significantly low as compared to HBV/C1 and HBV/D2 (Fig 2A). The levels of bilirubin followed a similar pattern as that of ALT; however the differences among different sub-genotypes were not significant (Fig 2B).

The HBV DNA load in our study population ranged from 2 log copies/ml to 5.95 log copies/ml with a median load of 2.13 log copies/ml. There was no significant variation of viral load among different HBV genotypes and sub-genotypes.

### Demographic data and HBV genotypes compared between Acute and Chronic Infections

**Fig 3:**
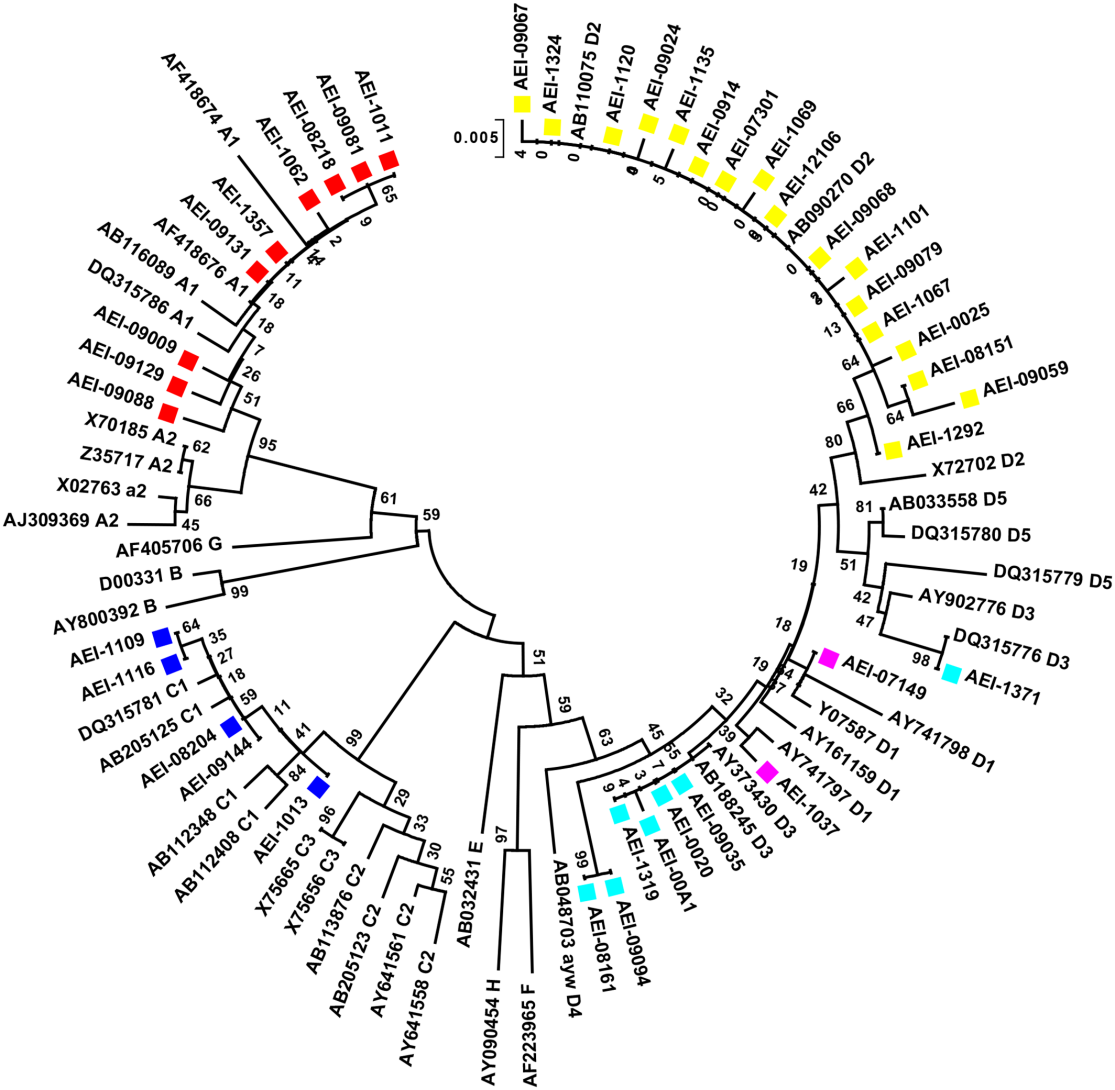
Phylogenetic analysis of HBV isolates from Eastern India with acute infection created using the neighbour-joining method. Phylogenetic tree was constructed from surface gene sequences of this study (denoted by AEI) along with reference sequences derived from GenBank (denoted by accession numbers).

**Fig 4:**
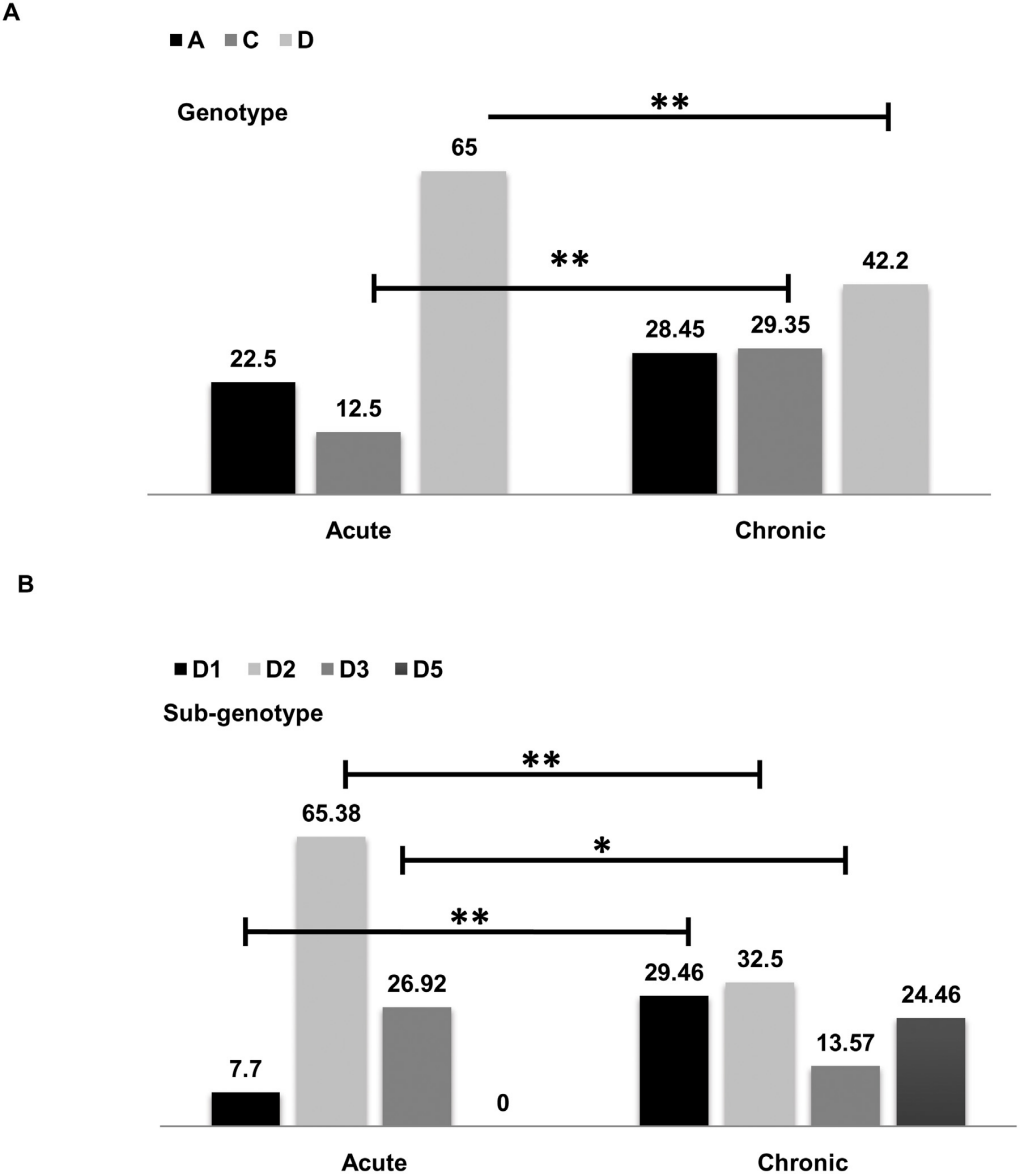
Distribution of HBV genotypes and sub-genotypes among acute and chronic patients of eastern India. (A) Difference in HBV genotype distribution among acute and chronic patients. (B) Difference in the distribution of HBV sub-genotypes under genotype D among acute and chronic patients. Only significant results (*p<0.05, **P<0.01) have been marked in the figure.

Demographic, phylogenetic and mutational patterns of acute patients of the current study were compared with already reported data and sequences of chronic patients who had visited our lab in the same time-frame [14, 15, 16, 17]. The chronic patients used were symptomatic as well as asymptomatic with mild to moderate liver inflammatory manifestations. However, data from patients with advanced Liver Disease including Liver cirrhosis or Hepatocellular carcinoma were excluded from the study. Table 1 displays the demographic data in acute patients as compared to chronic patients. There were significant differences in the percentage of affected males/females, HBeAg positivity, ALT and HBV viral DNA titer between acute and chronic infections. HBeAg positivity and ALT levels were significantly high in acute patients whereas viral DNA titer and proportion of affected males were significantly high in chronic patients (Table 1). The distribution of HBV genotypes and sub-genotypes among acute patients has been demonstrated in Fig 3. Comparison of HBV genotypes between acute and chronic infection has been shown in Fig 4A. HBV/D was the dominant genotype in both. However, percentage of subjects belonging to genotype D was significantly high in acute when compared to chronic patients whereas percentage of genotype C was significantly high in chronic patients as compared to acute. However, there was no significant difference in the frequency of genotype A in acute Vs chronic patients. Differences in HBV sub-genotypes have been demonstrated in Fig 4B. Interestingly, sub-genotypes under HBV/D showed considerable variability in acute patients when compared to chronic. Sub-genotype D2 was most prevalent among acute patients and was significantly high as compared to chronic patients (65.38% Vs 32.5%; p<0.01). Notably, HBV/D5 was absent in acute patients. Frequency of HBV/D1 and HBV/D3 also varied significantly in acute Vs chronic infection (Fig 4B).

**Table 1:** Demographic, biochemical and virological characteristics of acute and chronic subjects from eastern India. Statistically significant values are denoted by *(p<0.05) or **(p<0.001).

	Acute	Chronic
**No. of patients**	40	95
**Age**	33.43±12.86	32.72±11.8
**male%;female%**	70%; 30%	85.26% 14.74% *
**HBeAg positivity**	32.50%	18.94% **
**ALT (IU/liter)**	746.06±80.6	59.7 ±30.1**
**viral load(log copies/ml)**	2.72±1.04	4.52±0.75*

### Frequency of signature mutations in HBV genome compared in acute and chronic Infections

**Fig 5:**
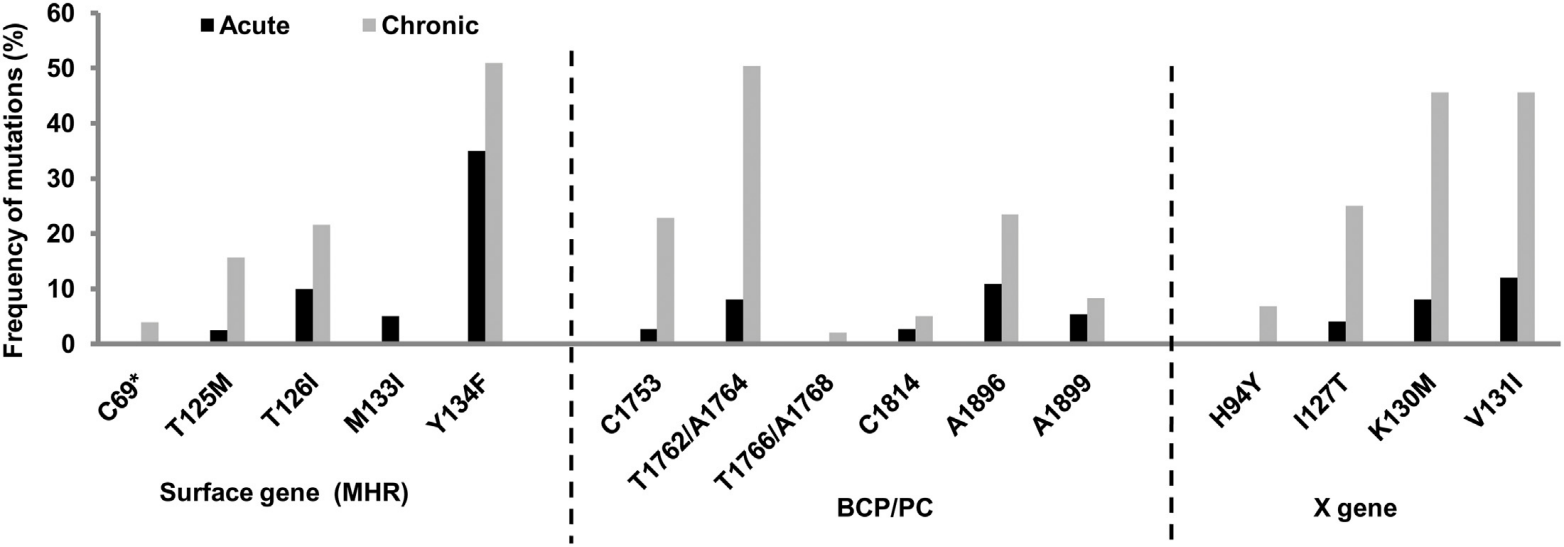
Frequencies of some principal mutations in the HBV genome compared in acute and chronic Hepatitis B patients. Figure depicts some signature mutations in the surface gene (S), basal core promoter/pre-core regions (BCP/PC) and X gene.

The mutations in the partial surface (S) gene region encompassing the MHL (major hydrophilic loop), the basal core promoter and pre-core (BCP/PC) region and the X gene were compared in acute and chronic infection (Fig 5). Of the 40 HBV DNA positive acute patients whose partial S gene could be amplified, the BCP/PC, whole small S gene and X gene could be amplified and sequenced in 37(92.5%), 30(75%) and 25(62.5%) isolates respectively. This was compared to 67 BCP/PC, 51 small S and 33 X gene sequences from chronic patients coming to our lab during this period. As compared to chronic infection, substitutions were relatively low in acute infection. Important mutations in the MHL of the surface gene that were detectable in acute hepatitis, were T125M (2.5%), T126I (10%), and Y134F (35%). The frequencies of these mutations in chronic infection were 15.69%, 21.56% and 50.9% respectively. The C69stop codon mutation was undetectable in acute infection. Interestingly, the substitution M133I was detectable in 2 isolates (5%) manifesting acute infection but was completely absent in all the chronic isolates. The substitutions in the polymerase region overlapping with the surface gene were also analyzed. There were no drug resistant mutations (lamivudine-resistant pattern: rtM204V/I, rtL180M, rtV173L, adefovir-resistant pattern: rtA181V/T, tenofovir-resistant pattern: rtA194T and entecavir-resistant pattern: rtL180M, rtS202G, rtM204V) detectable in acute patients belonging to our study population.

Major substitutions that were detectable in the BCP/PC region of acute isolates included T1753C (2.7%), A1762T/G1764A (8.1%), A1814C (2.7%), G1896A (10.8%) and G1899A (5.4%). Their respective frequencies in chronic infection were 22.78%, 50.445%, 5%, 23.445% and 8.22% respectively. The C1766T/T1768A double mutation was however detectable in only chronic infection (2%). Notably, the frequency of pre-core substitutions (A1814C, G1896A, and G1899A) was relatively high in acute infection reaching to almost half of that present in chronic infection.

Important substitutions in the X gene included I127T, K130M, and V131I whose frequencies in acute infection were 4%, 8% and 12% respectively and in chronic infection were 24.99%, 45.57%, 45.57% respectively. Substitution H94Y was absent in acute infection.

It is noteworthy, that the C69stop codon mutation in the surface gene, the C1766T /T1768A mutation in the basal core promoter and the H94Y mutation in the x gene were found only in chronic patients. However, we have found no significant association of these mutations with HBeAg negativity and ALT levels among chronic patients.

### Nucleotide diversity in the HBV genome compared in acute and chronic Infection

**Fig 6:**
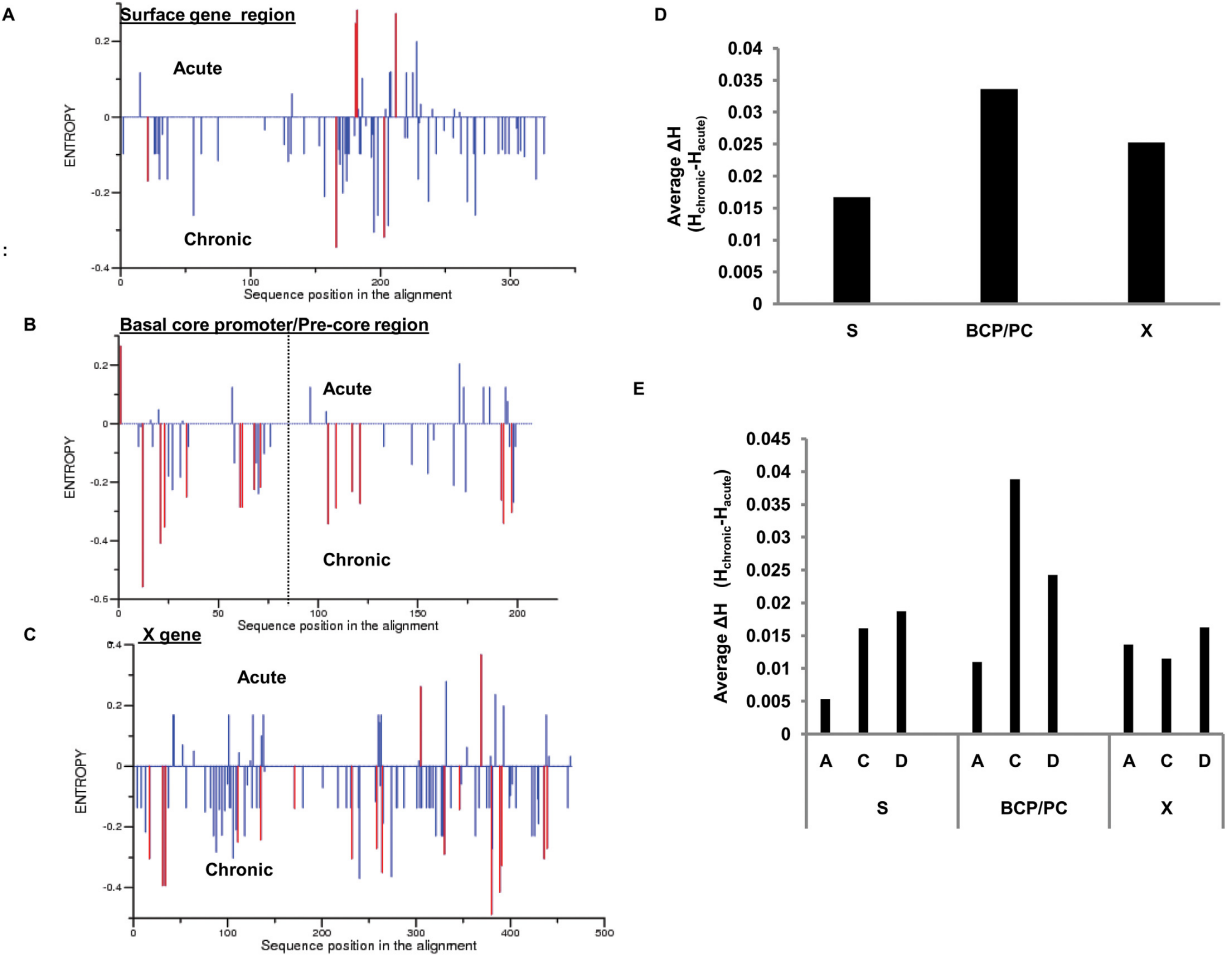
Comparison of nucleotide diversity in acute Vs chronic infection. Nucleotide diversity in the (A) surface gene, (B) BCP/PC region and (C) X gene has been compared. The nucleotide sequences of the chronic sequences were compared to that of the acute sequences using Shannon’s Entropy. The blue lines indicated the specific positions where changes were noted. The red lines indicate the positions where the frequency of substitution between acute and chronic sequences was significantly different. (D) The average ΔH (average difference in entropy between chronic and acute infection) was highest in the BCP region of the HBV genome. (E) Calculation of genetic diversity at the genotype level showing that the BCP/PC region of genotype C isolates exhibit highest average ΔH.

To identify the nucleotide diversity, the nucleotide sequences of the chronic isolates were compared with that of the acute isolates by Shannon’s Entropy (Fig 6A, 6B and 6C). The partial S gene region, the BCP/PC region and the X gene were used for this analysis. In totality, the diversity was considerably higher in chronic sequences as compared to acute in all these regions. The average ΔH (average difference in entropy between chronic and acute infection) in the surface gene, BCP/PC region and X gene were 0.01676, 0.03364 and 0.02529 respectively. Clearly, there was a conspicuous difference in the nucleotide diversity in all these regions. However the highest average ΔH was observable in the BCP/PC region of the HBV genome (Fig 6D). The difference in entropy between chronic and acute sequences were analyzed at the genotype level in each of the concerned regions (i.e. S, BCP/PC and X gene). This revealed that the BCP/PC regions of especially genotype C isolates, exhibited the highest ΔH (Fig 6E). The nucleotide positions those were most vulnerable to acquiring mutations during HBV persistence i.e. significantly high frequency in chronic were estimated from Randomization results excluding genotype specific signatures. We noted significant changes at position 528 (C→T) (amino acid substitution T125M) in the S gene and positions 1753 (T→C), 1762(A→T), 1764(G→A), 1933 (T→G), and 1938 (T→C) in the BCP/PC region. No such changes were found in the X gene except at positions overlapping with the BCP (amino acid substitutions T127I, M130K and I131V). However among them the highest difference in entropy was exhibited at positions 1762 and 1764 (ΔH = 0.409 and 0.353 respectively; p<0.05) in the BCP.

## Discussion

The current study presents a comprehensive picture of HBV strains circulating among acute Hepatitis patients in Eastern India. The acute HBV strains were compared with chronic HBV strains in an attempt to find out the key factors that are responsible for viral persistence. To the best of our knowledge, this is the first study from India to characterize HBV strains of acute hepatitis patients at the molecular level.

We have stringently scrutinized the patients before enrolling them for the present study. Only patients with high levels of antiHBc-IgM and history of HBV infection for less than 6 months were included in the study in order to eliminate patients with acute exacerbation of chronic infection [19]. Patients without clear medical records were excluded. After a rigorous screening of 653 HBsAg positive patients only 40 (6.12%) were found to have acute HBV infection. This is quite low in comparison to a report from China, where 182 of 507 (35.9%) were found to have acute HBV infection [20]. This might be because HBV is highly endemic in China whereas India is placed in the intermediate HBV endemicity zone. Another report from Argentina also shows a considerable prevalence of acute infection (28.20%) among HBsAg positive patients [21]. This can be justified by a previous report which affirms that the occurrence of acute as well as fulminant hepatitis is high in western countries in comparison to the eastern world where chronic infection dominates [3].

Our examination on the modes of transmission revealed that a vast majority of the infected patients (55%) were unaware of their source of infection calling for an urgent need of HBV awareness programs. However, some of them also include subjects who refused to disclose their transmission route. Patients may be hesitant to reveal sexually promiscuous behavior or use of injectable drugs due to social stigma. Of the rest (whose sources of infection were known), sexual promiscuity emerged as the predominant mode by which HBV is transmitted in our study group. An earlier report from South Italy also acknowledges sexual transmission to be the major route by which acute HBV is transmitted [22]. In contrast, a report from China illustrates that pedicure in bath centers exceeds all other risk factors in transmission of acute infection. Though our finding is limited by the sample size, we extended our analysis with patients’ age groups which disclosed that the age bracket in between 15–30 years showed the highest frequency of HBV occurrence. Interestingly this accounts for the most sexually active age group. The frequency of HBV occurrence decreased with an increase in age for the same reason. This calls for the need to emphasize on various HBV awareness programs in schools, colleges, offices, etc. so as to address this vulnerable population. Nevertheless, an initiation of adolescent vaccination strategies can be helpful in the long term to prevent fresh HBV infections. Several programs in India, organized to increase the relative awareness on HIV infection among the general population have been very effective in raising a common consciousness and in the long term in bringing down the HIV occurrence in totality (NACO) (http://www.ndtv.com/article/india/world-aids-day-india-records-sharp-drop-in-number-of-cases-299730). HBV is 50–100 times more infectious than HIV (UNCDC). In spite of this, social awareness of HBV is extremely limited in India. Initiation of universal HBV vaccination curriculum among infants has been a commendable step in India [23]. Instigation of adolescent vaccination can further help in serving the purpose.

Our study clearly illustrates a distinctive difference in genotype distribution between acute and chronic HBV infection. We report a dominance of HBV/D in acute patients which is in concordance with some earlier reports from the western world like the US and Italy [3, 24]. However, in some Asian countries, such as China and Korea, genotype C predominates among acute HBV patients [5, 25]. On the other hand, in the present study, HBV/C was significantly higher in chronic when compared to acute, thus affirming that HBV/C is more associated with chronic infection in India as compared to HBV/A and HBV/D, the other two genotypes circulating among patients from India. This report is in accordance with most other studies from India and elsewhere establishing genotype C to be associated with end-stage liver disease including Liver cirrhosis and hepatocellular carcinoma [26, 27, 28, 29].

HBV/ D, the predominant genotype in India is further divisible into a number of sub-genotypes, of which HBVD1, HBVD2, HBVD3 and HBVD5 are found in India [29]. The present study displays a conspicuous difference in the distribution pattern of these sub-genotypes between acute and chronic HBV infected patients. Though HBV/D2 is predominant in both cases, its frequency in acute infection is remarkably high. Interestingly, studies conducted with patients co-infected with HBV and HIV belonging to the same geographical area also shows the presence of a high frequency of HBV/D2 [30]. This might be because of the similarity in the routes of transmission, thus further establishing the association of HBV/D2 with that of sexual exposure.

Further analysis of HBV genotypes/sub-genotypes revealed that prevalence of HBV sub-genotypes tends to vary with different modes of HBV transmission. Among patients who admitted to sexually promiscuous behavior, sub-genotypes HBV/D2 & HBV/D3 were found to be most prevalent. In contrast, patients who claim to have been infected via surgeries or blood transfusion were associated with a variety of subgenotypes. This could possibly be explained by the fact that surgery and transfusion associated patients were exposed to multiple sources of infection.

Our analyses of the mutational patterns of the S, BCP, PC, and X gene regions reveal that the frequencies of most of the mutations are higher in chronic patients as compared to acute. Among them the C69stop codon mutation in the surface gene, the C1766T /T1768A mutation in the basal core promoter and the H94Y mutation in the x gene were found only in chronic patients showing its clear association with chronicity. Interestingly, the frequency of the mutation G1896A in the pre-core region was relatively high among acute patients almost reaching to about half of the number found among chronic patients, thus revealing that this mutation is highly transmissible. Earlier reports [29] have found an association of this mutation with end stage liver disease. The M133I substitution in the major hydrophilic region of the surface gene is a known immune escape mutation [31, 32]. Though this mutation has been found in some other isolates from HBV mono-infected patients, its frequency in patients co-infected with HIV is relatively high [13, 30, 33, 34,]. Notably, the M133I mutation was found in 2 acute isolates, but was missing from all chronic sequences indicating that this mutation might be a recent introduction into our population. Since this mutation is an established immune escape mutation, its introduction to the study population is noteworthy. A recent report from China showed that 7% of their acute HBV patients carried drug resistant mutations [4]. But our analysis of the polymerase region overlapping with the S gene did not reveal the presence of such mutations.

The nucleotide diversity of the partial S gene region, the BCP/PC region and the X gene were evaluated as these are the most polymorphic sites in the HBV genome. Comparison of nucleotide diversity of these regions among acute and chronic patients revealed that the BCP/PC region shows the highest average ΔH indicating that this region is most vulnerable to acquiring mutations upon viral persistence. The BCP/PC regions are associated with the regulation and expression of hepatitis B e antigen (HBeAg). Though HBeAg is not necessary for viral replication, it appears to be essential for establishing viral persistence in animal models [35]. Again, we have found that in the BCP/PC region, genotype C isolates presented the highest average ΔH reflecting its increased tendency towards acquiring mutations and heading towards chronic infection. The association of HBV/C with BCP mutations has been demonstrated in a number of early studies [36, 37]. Moreover, we report that the highest difference in entropy was exhibited at positions A1762T and G1764A in the BCP region indicating that these two sites were most susceptible towards acquiring mutations, thus signifying that they were the strongest indicators of chronicity. Reports specify that the presence of BCP mutations affecting HBeAg expression during the acute stage is associated with more severe clinical paths and/or fulminant hepatic failure [38, 39, 40, 41, 42] which emphasizes the need to screen for BCP/PC mutations in infected acute patients.

In conclusion, our current study documents diverse HBV strains circulating among acute hepatitis patients in Eastern India whereby HBV/D2 is most prevalent and circulates primarily through sexual routes. Drug resistant mutations were not detected in the polymerase gene of acute patients. Additionally, we also screened for the region and nucleotide positions that commonly acquire mutations upon HBV persistence. Our study thus underscores the need for an appropriate preventive strategy as well as identifies mutations that might be related to the possibility of progression to chronic infection.
